# Treatment quality and outcome for multidrug-resistant tuberculosis patients in four regions of China: a cohort study

**DOI:** 10.1186/s40249-020-00719-x

**Published:** 2020-07-18

**Authors:** Xu-Bin Zheng, Vinod K. Diwan, Qi Zhao, Yi Hu, Judith Bruchfeld, Wei-Li Jiang, Sven Hoffner, Biao Xu

**Affiliations:** 1 Department of Epidemiology, School of Public Health and Key Laboratory of Public Health Safety (Ministry of Education), Fudan University, 130 Dong An Road, Shanghai, 200032 China; 2 Department of Global Public Health, Karolinska Institutet, Stockholm, Sweden; 3 Department of Infectious Diseases, Karolinska University Hospital, Stockholm, Sweden; 4 Division of Infectious Diseases, Department of Medicine Solna, Karolinska Institutet, Stockholm, Sweden

**Keywords:** Multidrug-resistant, Tuberculosis, Delay, Treatment, Follow-up, China

## Abstract

**Background:**

China incurs an extremely low treatment coverage of multidrug-resistant tuberculosis (MDR-TB). This study aimed to understand the experience of MDR-TB patients on quality of health care, and the clinical impact through an up to six-year follow-up.

**Methods:**

Cohorts of MDR-TB patients were built in TB/MDR-TB designated hospitals in four regions of China from 2014 to 2015. Patients were followed up during treatment course, and yearly confirmation afterward until 2019. Delay in MDR-TB diagnosis and treatment was calculated upon bacteriological confirmation and treatment initiation. Risk factors for unfavourable outcomes were identified by multivariate logistic regression.

**Results:**

Among 1168 bacteriological-positive TB patients identified from a 12-million population, 58 (5.0%) MDR-TB cases were detected. The median delay for MDR-TB diagnosis was 90.0 days, with 13.8% having a delay above 180.0 days. MDR-TB treatment was only recommended to 19 (32.8%) participants, while the rest continued with regimen for drug-susceptible TB. In MDR-TB treatment group, 36.8% achieved treatment success, while the others had incomplete treatment (21.1%), loss to follow-up (36.8%) and TB relapse (5.3%). For non-MDR-TB treatment group, 33.3% succeeded, 25.6% relapsed, 2.6% failed, 23.1% died, and 15.4% were lost to follow-up. Overall, only 35.7% (20/56) of detected MDR-TB patients had favourable outcomes and higher education level was positively associated with it (adjusted odds ratio [a*OR*]: 3.60, 95% confidence interval [*CI*]: 1.04–12.5).

**Conclusions:**

A large proportion of patients did not receive MDR-TB treatment and had unfavourable outcomes. Delayed MDR-TB diagnosis resulted in poor quality of MDR-TB care. Rapid diagnosis, regulated patient management and high-quality MDR-TB treatment should be enhanced in China.

## Background

Multidrug-resistant tuberculosis (MDR-TB) has become a global public health crisis, threatening the achievement of “Ending the global TB epidemics” in 2035 [1]. According to the World Health Organization (WHO) Global TB report [2], there were an estimated 500 000 new cases having MDR/rifampicin-resistant (RR)-TB in 2018, with an annual increase of over 20% between 2009 and 2016 [1]. Half of the MDR/RR-TB burden was laid on three countries, i.e., India, China, and the Russian Federation [2]. To fight MDR-TB, globally five priority actions have been proposed, i.e., providing high-quality treatment of drug-susceptible TB, scaling up rapid testing and detection of MDR/RR-TB, ensuring prompt access to effective treatment and proper care, minimizing the risk of disease transmission by quickly enrolling diagnosed patients on effective treatment, and increasing political commitment to ensure necessary financing [3].

China has implemented the modern TB control program since the 1990s, featured by the directly observed treatment, short course strategy (DOTS). With political commitment and pro-poor policy, morbidity and mortality of TB in China have been continuously decreasing at an annual drop of 2.32% and 7.77% respectively, from 2000 to 2013 [4]. Although great achievements have been made, China remains the country having the second highest burden of TB and MDR-TB in the world [2]. The challenges to China’s MDR-TB control are not only the heavy disease burden but also the poor accessibility and quality of MDR-TB care [2, 5, 6]. As the WHO reported in 2018, the MDR-TB detection rate was only 22.8% in China coupled with a treatment coverage of 13.6% [2]. Poor accessibility to drug susceptibility testing (DST) especially in rural areas [7], long waiting time for conventional DST results [8] and incomplete high-risk screening strategy for MDR-TB [9] might explain the low detection rate of MDR-TB in China; while delayed DST, unaffordable treatment cost, drug-induced adverse effects and fragile supply chain of second-line drugs could be the main reasons for the low treatment coverage [5, 10, 11].

Although several studies reported the difficulties in accessing to MDR-TB diagnosis [7, 12], few have directly illustrated what patients experienced after they were diagnosed as MDR-TB, for instance, what kind of treatment they received and how the quality of care influenced their treatment outcomes. MDR-TB patients should be provided with a recommended regimen containing at least five effective second-line anti-TB drugs in a 6-month intensive phase and at least four drugs in an 18-month consolidation phase according to the national MDR-TB prevention and control program [13], or a standardized shorter MDR-TB regimen as recommended recently by the WHO [14]. Using these guidelines, treatment outcomes can only be assessed in patients who have actually initiated the recommended treatment course. The outcomes of those who did not receive MDR-TB treatment and those treated but lost to follow-up, having incomplete or failed treatment were also critically important from a TB control program perspective. This study was carried out in a cohort of MDR-TB patients in China. The aims of the study were to illustrate the experiences of MDR-TB patients on diagnosis and treatment under China’s MDR-TB control program, to identify problems in the provision of high-quality MDR-TB care, and to understand the association between quality of care and treatment outcomes with considerations of demographic and clinical characteristics of patients and molecular features of infected *Mycobacteria tuberculosis (M.tb)*.

## Methods

### Study design

As a prospective follow-up study, a cohort of MDR-TB patients was conducted in TB/MDR-TB designated hospitals in four geographically varied provinces in China, with two located in east and two in west. The study sites were chosen with considerations of geographic variation, economic level, healthcare infrastructure and TB epidemic status. The population for selected four study sites was 2.0, 3.8, 2.7 and 3.5 million in 2014, respectively. Basic TB care is designated in county or district TB clinics, where sputum smear microscopy is provided for TB diagnosis. Sputum culture was performed for all smear-positive TB patients and for smear-negative TB patients with typical pulmonary lesions on chest X-ray. All positive cultures were submitted to the up-level laboratory in the prefectural Center for Disease Control and Prevention (CDC) or TB hospital for DST, mostly, the conventional DST.

Once the MDR-TB diagnosis was made, patients should be transferred to the designated prefectural TB hospitals for diagnosis confirmation, MDR-TB treatment and patient management. Patients aged > 18 years, confirmed with MDR-TB between January 2014 and December 2015, and providing written inform consent were enrolled in this study. Clinical follow-up was given by clinicians in the designated TB hospitals monthly during the intensive phase, and every other month during the consolidation phase. Additional yearly contact was given from 2014 to 2019 by the healthcare workers in the CDC to follow up patients’ TB status and progress.

### Microbiological analyses

Three sputum samples from each patient were collected for direct smear microscopy. Mycobacterial culture was subsequently given to those with positive sputum smear, and those with negative smear but typical pulmonary lesions on chest X-ray, using Loewenstein-Jenson (LJ) medium. Species identification and DST were performed for all culture-positive *M.tb* isolates. Rifampicin, isoniazid, ethambutol, streptomycin, one fluoroquinolone (ofloxacin or levofloxacin) and one second-line injectable drug (kanamycin, capreomycin or amikacin) were included for DST using the proportion method on LJ medium with the following critical concentrations: 40.0, 0.2, 2.0, 4.0, 2.0, 2.0, 20.0, 10.0, and 4.0 mg/L, respectively [15].

### Definitions of treatment regimen and outcomes

MDR-TB treatment was defined as receiving recommended regimen in the national MDR-TB control program [13]. Non-MDR-TB treatment in this study referred to first-line TB treatment for drug-susceptible TB, which patients had taken before diagnosed as MDR-TB, and was kept unchanged after the diagnosis. MDR-TB diagnosis delay was defined as the time period from the diagnosis of smear-positive TB to MDR-TB. TB/MDR-TB treatment delay was the time period between diagnosis of smear-positive TB/MDR-TB and initiation of TB/MDR-TB treatment. The definition of treatment outcomes depended on the treatment regimen patients received [16]. Cure and treatment completion were defined as favourable outcomes while loss to follow-up, failure, relapse, death and incomplete treatment were uniformly called unfavourable outcomes.

### Genotyping and gene sequencing

Cetyl Trimethyl Ammonium Bromide (CTAB) method was applied to extract DNA from baseline *M.tb* isolates. Spoligotyping was used to identify the families of the strains and the phylogenetic clades were assigned with reference to SpolDB4 database. Previously reported drug resistance-determining loci [17], including *gyrA, gyrB, rrs, eis and pncA*, were sequenced and mapped to the sequences of published *M.tb* reference strain H37Rv to identify the presence or absence of mutations.

### Data collection and statistical analysis

A structured questionnaire was applied to collect socio-demographic and clinical information of participants at the time of TB diagnosis, including age, gender, image of chest X-ray, previous TB treatment history and comorbidities. Details of treatment regimen, sputum culture, DST results, clinical progresses, dates of testing and reporting, and treatment course were extracted from patient’s medical chart. After the end of treatment course, yearly follow-up was given to confirm the health condition and TB status. In addition, information in the national TB registration system was exported and matched to the study participants to confirm the TB relapse. Chi-square test and Mann-Whitney U test were applied to identify the differences of socio-demographic and clinical characteristics between patients receiving MDR-TB or non-MDR-TB treatment. Univariate binary logistic regression was used to analyse the factors associated with treatment outcomes, while age, gender and study sites were used for adjustment in multivariate analysis. All statistical analyses were performed with IBM SPSS 22.0 (IBM Corp., Armonk, NY).

## Results

### Study patients

In total, 1168 bacteriological-positive TB patients, including 1084 smear-positive and 84 smear-negative while culture-positive TB patients, were diagnosed over the population of 12 million from 2014 to 2015. Of them, 58 (5.0%) were later confirmed as MDR-TB. The mean age of MDR-TB patients was 45.4 years and 84.5% of them were male. There were 19 (32.8%) patients who were transferred to MDR-TB care, while the other 39 (67.2%) were still under treatment for drug susceptible TB (non-MDR-TB treatment group), although 5.1% (2/39) received an additional fluoroquinolone (FQ). The reasons for not transferring to MDR-TB treatment were: already taking the drug-susceptible TB treatment for a couple of months, effective therapeutic effects judged by clinicians or refusal made by patients. Compared to patients with MDR-TB but not on appropriate treatment, those receiving MDR-TB treatment were younger, to a larger proportion of females and from eastern areas, and with a lower coverage of medical insurance (*P* < 0.05), including two patients under a local pro-poor MDR-TB project. (Table 1).

**Table 1:** Demographic, clinical and molecular characteristics of MDR-TB patients and isolated *Mycobacterium tuberculosis* strains ^a^

Factors	All patients (*n* = 58)	Treatment regimen		*P* value
		Non-MDR-TB (*n* = 39)	MDR-TB (*n* = 19)	
Age ^b^	45.4 ± 17.3	49.6 ± 17.6	36.8 ± 13.4	0.009
Male	49 (84.5)	36 (92.3)	13 (68.4)	0.047
Study sites in				
Western	41 (70.7)	31 (79.5)	10 (52.6)	0.035
Eastern	17 (29.3)	8 (20.5)	9 (47.4)	
Body mass index ^b^	20.4 ± 2.3	20.7 ± 2.3	19.8 ± 2.2	0.191
Education				
Primary school or below	38 (65.5)	25 (64.1)	13 (68.4)	0.745
Junior high school or above	20 (34.5)	14 (35.9)	6 (31.6)	
Married	31 (53.4)	20 (51.3)	11 (57.9)	0.636
Health insurance	51 (87.9)	38 (97.4)	13 (68.4)	0.004
Haemoptysis	15 (25.9)	11 (28.2)	4 (21.1)	0.752
Tuberculosis treatment history	25 (43.1)	19 (48.7)	6 (31.6)	0.216
Pulmonary cavities	28 (48.3)	17 (43.6)	11 (57.9)	0.306
DST pattern				
MDR	31 (53.4)	17 (43.6)	14 (73.7)	0.031
Pre-XDR and XDR	27 (46.6)	22 (56.4)	5 (26.3)	
Genotypic drug resistance (*n* = 52)				
*katG/inhA* mutation	38 (73·1)	22 (62·9)	16 (94·1)	0·021
*rpoB* mutation	49 (94·2)	32 (91·4)	17 (100·0)	0·542
*gyrA/gyrB* mutation	20 (38·5)	14 (40·0)	6 (35·3)	0·744
*rrs/eis* mutation	8 (15·4)	7 (20·0)	1 (5·9)	0·248
*pncA* mutation	14 (26·9)	11 (31·4)	3 (17·6)	0·341
Strain family (*n* = 52)				
Non-Beijing family	6 (11.5)	4 (11.4)	2 (11.8)	1.000
Beijing family	46 (88.5)	31 (88.6)	15 (88.2)	

DNA was failure to extract from six *Mycobacterium tuberculosis* isolates. *MDR* Multidrug-resistant, *DST* Drug susceptibility testing, *XDR* Extensively drug-resistant

^a^Data are presented as number (percent) unless other specifies

^b^Continuous variable; means ± standard deviations are presented

### Drug resistance and strain families

The DST results showed that 31 (53.4%) *M.tb* isolates were resistant to isoniazid and rifampicin without additional resistance, 17 (29.4%) and 5 (8.6%) isolates had additional resistance to FQs or second-line injectable drugs (SLID), i.e. pre-extensively drug-resistant (pre-XDR), while the remaining five (8.6%) isolates were resistant to both FQs and SLID and thus defined as XDR. The consistencies between phenotypic and genotypic drug resistance were 73.1% on isoniazid and 94.2% on rifampicin. Of the 22 FQs-resistant strains, 16 (72.7%) were detected with mutations in *gyrA/gyrB* loci, while mutations in *rrs/eis* loci were observed in 75.0% (6/8) of SLID-resistant strains. Results of spoligotyping showed that 46 (88.5%) *M.tb* strains belonged to Beijing family. It was found that non-MDR-TB treatment group had higher proportion of pre-XDR and XDR, and lower proportion of mutations in *katG/inhA* loci, compared to the group transferred to MDR-TB treatment (*P* < 0.05) (Table 1).

### Diagnosis and treatment delay

**Fig. 1:**
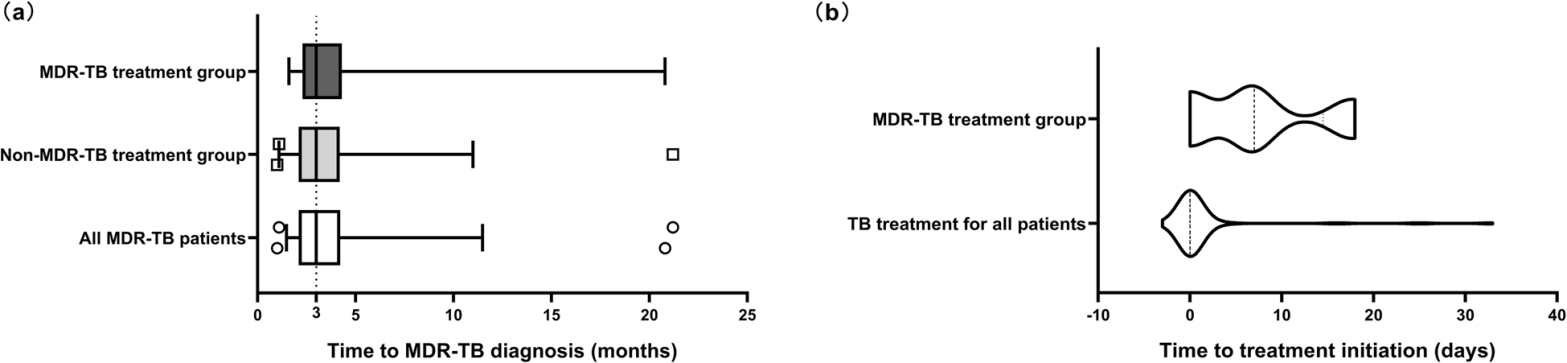
Time to multidrug-resistant tuberculosis (MDR-TB) diagnosis and treatment initiation. **a** Time period from diagnosis of a smear-positive TB to MDR-TB in all MDR-TB patients, patients receiving recommended MDR-TB treatment, and patients continued with the treatment for drug-susceptible TB, namely non-MDR-TB treatment. **b** Time period from the diagnosis of smear-positive TB to the initiation of TB treatment in all patients, and time period from the diagnosis of MDR-TB to the initiation of the recommended MDR-TB treatment in those transferred to MDR-TB treatment

The median delay for MDR-TB diagnosis was 90.0 (interquartile range [IQR]: 64.0–127.5) days with no significant differences between MDR-TB and non-MDR-TB treatment groups (*P* > 0.05). Of all participants, 51.7% (30/58) had a diagnosis delay over 90.0 days and 13.8% (8/58) had a delay even over 180.0 days. In terms of treatment delay, the non-MDR-TB treatment group kept their regimen for drug-susceptible TB while the MDR-TB treatment group had a median delay for 7.0 (IQR: 0.0–14.5) days before initiating the recommended treatment. The total delay, summed by diagnosis and treatment delay, for the MDR-TB treatment group was 91.5 (IQR: 83.8–134.0) days (Fig. 1).

### Treatment outcomes

In the non-MDR-TB treatment group, 28 (71.8%) patients met the requirements for “treatment success” while the rest had treatment failure, death or loss to follow-up at the end of treatment course. In the group transferred to MDR-TB treatment, only 8 (42.1%) patients had completed their MDR-TB treatment and 7 succeeded. Overall, 71.8% (28/39) of patients in non-MDR-TB treatment group and 36.8% (7/19) of patients in MDR-TB treatment group were reported as having favourable outcomes (*P* < 0.05) at the end of their administrated treatment course.

**Fig. 2:**
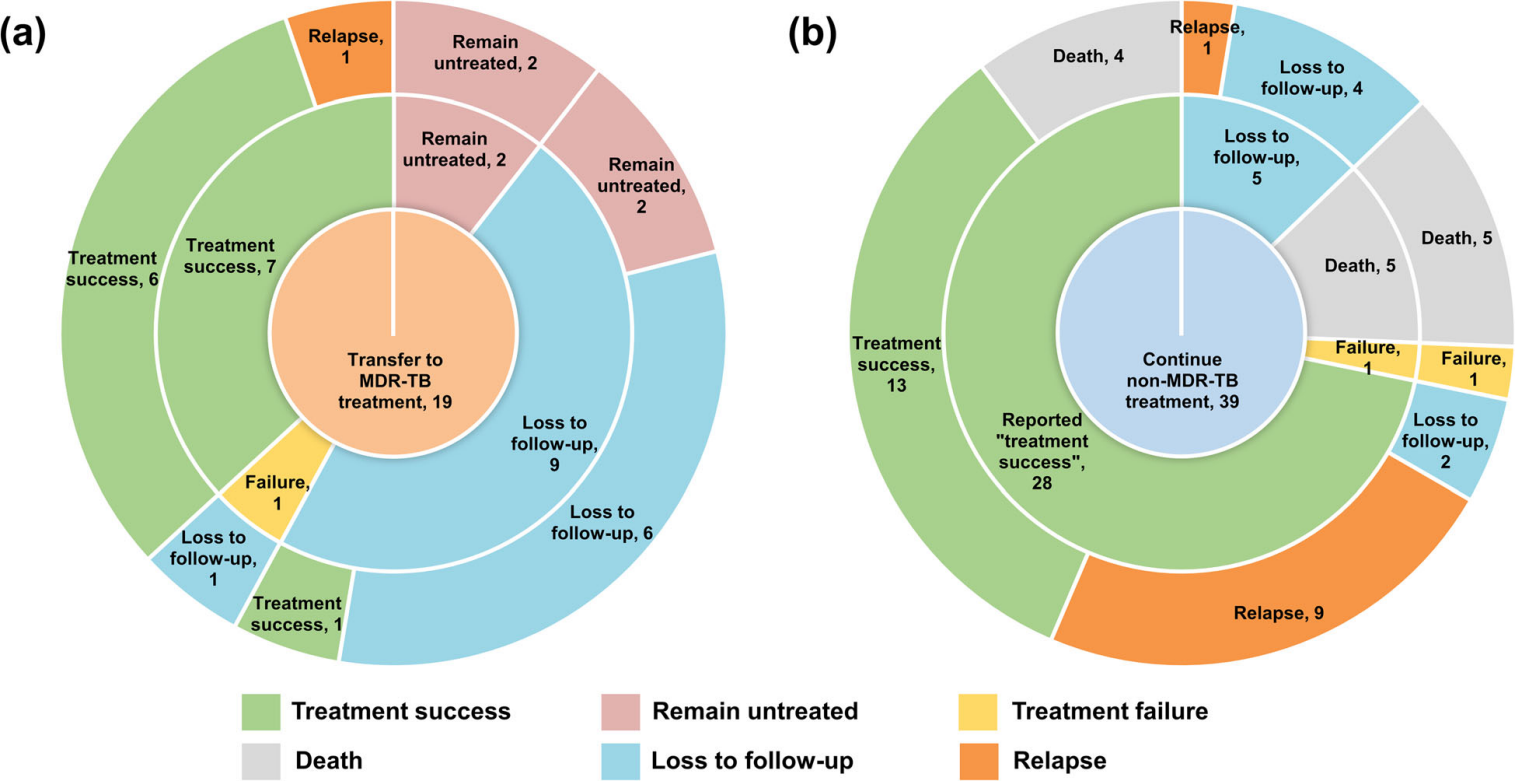
Treatment outcomes for participating multidrug-resistant tuberculosis (MDR-TB) patients receiving MDR-TB treatment (**a**) and non-MDR-TB treatment (**b**). The circles from inside to outside represented treatment regimen, treatment outcomes at the end of treatment course, and treatment outcomes at the last follow-up in 2019

Further yearly follow-up until the end of 2019 disclosed that in the group kept drug-susceptible TB treatment (*n* = 39), only 13 (33.3%) patients remained “treatment success”; 10 (25.6%) had TB relapse, including 9 previous successfully treated patients; another 4 (10.3%) died after reported as “treatment success”, of which 50% had a main cause of death as TB; 1 (2.6%) did not receive further treatment after failing the previous treatment; and 6 (15.4%) were lost to follow-up, of which 4 did not complete their treatment and 2 were reported as “treatment success”. In the group transferred to MDR-TB treatment, 7 (36.8%) had treatment success upon follow-up; 1 (5.3%) had a TB relapse after being successfully treated; 4 (21.1%) remained untreated, including 2 identified from the loss to follow-up group; and 7 (36.8%) were lost to follow-up. The treatment success rate among patients who actually completed their treatment was 46.4% (13/28) in non-MDR-TB treatment group, while it was 77.8% (7/9) in MDR-TB treatment group. Overall, favourable outcomes were decreased from the previous 71.8% (28/39) to 35.1% (13/37) in patients with non-MDR-TB treatment, while remained to be 36.8% (7/19) in those transferred to MDR-TB treatment at the last follow-up. The median time for TB relapse after treatment completion was 24.0 (IQR: 12.8–32.5) months and over 90% occurred within three years (Fig. 2).

### Factors associated with treatment outcomes

Risk factors for unfavourable treatment outcomes at final follow-up were analysed by univariate logistic analysis and followed by multivariate analysis with adjustment of age, gender and study sites. Two patients from non-MDR-TB treatment group were excluded from analysis due to loss to follow-up after being reported as “treatment success” at the end of treatment course. In Table 2, it showed that patients with higher education level were more likely to achieve favourable outcomes (adjusted odds ratio [a*OR*]: 3.60, 95% confidence interval [*CI*]: 1.04–12.5). Such association was also observed in non-MDR-TB treatment group (a*OR*: 9.78, 95% *CI*: 1.77–54.0) (Table 3).

**Table 2:** Univariate and multivariate analysis of risk factors for unfavourable treatment outcomes among MDR-TB patients

Factors	Treatment outcomes at the last follow-up		Odds ratio (95% *CI*)	Adjusted odds ratio (95% *CI*) ^a^
	Favourable outcomes (*n* = 20)	Unfavourable outcomes (*n* = 36)		
Age (mean±SD)	40.8 ± 16.8	49.1 ± 16.9	0.97 (0.94–1.00)	
Male	16 (80.0)	31 (86.1)	0.65 (0.15–2.74)	
Study sites in				
Western	13 (65.0)	27 (75.0)	1	
Eastern	7 (35.0)	9 (25.0)	1.62 (0.49–5.30)	
Body mass index (mean±SD)	20.9 ± 2.0	20.0 ± 2.4	1.22 (0.94–1.58)	1.31 (0.99–1.75)
Education				
Primary school or below	9 (45.0)	28 (77.8)	1	1
Junior high school or above	11 (55.0)	8 (22.2)	4.28 (1.31–13.9)	3.60 (1.04–12.5)
Married	13 (65.0)	17 (47.2)	2.08 (0.67–6.41)	1.66 (0.50–5.55)
Health insurance	19 (95.0)	30 (83.3)	3.80 (0.42–34.1)	4.54 (0.49–42.0)
Haemoptysis	4 (20.0)	11 (30.6)	0.57 (0.15–2.10)	0.59 (0.15–2.29)
Tuberculosis treatment history	6 (30.0)	18 (50.0)	0.43 (0.13–1.36)	0.43 (0.13–1.49)
Pulmonary cavities	12 (60.0)	15 (41.7)	2.10 (0.69–6.39)	1.92 (0.52–7.12)
DST pattern				
MDR	12 (60.0)	18 (50.0)	1	1
Pre-XDR and XDR	8 (40.0)	18 (50.0)	0.67 (0.22–2.02)	0.87 (0.27–2.84)
Strain family (*n* = 50)				
Non-Beijing family	2 (10.5)	4 (12.9)	1	1
Beijing family	17 (89.5)	27 (87.1)	1.26 (0.21–7.64)	1.58 (0.24–10.4)
Treatment regimen				
Non-MDR-TB treatment	13 (65.0)	24 (66.7)	1	1
MDR-TB treatment	7 (35.0)	12 (33.3)	1.08 (0.34–3.40)	0.52 (0.13–2.10)
MDR-TB diagnosis delay (days)				
≤ 90.0	13 (65.0)	18 (50.0)	1	1
> 90.0	7 (35.0)	18 (50.0)	0.54 (0.17–1.66)	0.44 (0.12–1.55)

DNA was failure to extract from six *Mycobacterium tuberculosis* isolates. Two patients were once reported as treatment success but were lost at the last yearly follow-up, thus excluded from the analysis. *MDR* Multidrug-resistant, *SD* Standard deviation, *DST* Drug susceptibility testing, *XDR* Extensively drug-resistant

^a^Adjusted by age, gender and study sites

**Table 3:** Risk factors for unfavourable treatment outcomes among the MDR-TB patients received non-MDR-TB treatment

Factors	Patients with non-MDR-TB treatment		Odds ratio (95% *CI*)	Adjusted odds ratio (95% *CI*) ^a^
	Favourable outcomes (*n* = 13)	Unfavourable outcomes (*n* = 24)		
Age (mean±SD)	43.2 ± 19.8	55.1 ± 14.1	0.96 (0.91–1.00)	
Male	12 (92.3)	22 (91.7)	1.09 (0.09–13.3)	
Study sites in				
Western	10 (76.9)	20 (83.3)	1	
Eastern	3 (23.1)	4 (16.7)	1.50 (0.28–8.04)	
Body mass index (mean±SD)	21.4 ± 2.2	20.1 ± 2.2	1.33 (0.95–1.86)	1.41 (0.97–2.06)
Education				
Primary school or below	4 (30.8)	20 (83.3)	1	1
Junior high school or above	9 (69.2)	4 (16.7)	11.3 (2.29–55.4)	9.78 (1.77–54.0)
Married	8 (61.5)	11 (45.8)	1.89 (0.48–7.49)	1.69 (0.32–8.93)
Health insurance	13 (100.0)	23 (95.8)		
Haemoptysis	3 (23.1)	8 (33.3)	0.60 (0.13–2.81)	0.53 (0.10–2.84)
Tuberculosis treatment history	4 (30.8)	14 (58.3)	0.32 (0.08–1.33)	0.15 (0.02–1.05)
Pulmonary cavities	7 (53.8)	9 (37.5)	1.94 (0.49–7.64)	1.86 (0.37–9.19)
DST pattern				
MDR	7 (53.8)	9 (37.5)	1	1
Pre-XDR and XDR	6 (46.2)	15 (62.5)	0.51 (0.13–2.02)	0.64 (0.14–2.84)
Strain family (*n* = 33)				
Non-Beijing family	2 (16.7)	2 (9.5)	1	1
Beijing family	10 (83.3)	19 (90.5)	0.53 (0.06–4.32)	0.90 (0.08–10.2)
Total delay (days)				
≤ 90.0	9 (69.2)	12 (50.0)	1	1
> 90.0	4 (30.8)	12 (50.0)	0.44 (0.11–1.85)	0.36 (0.05–2.39)

DNA was failure to extract from six *Mycobacterium tuberculosis* isolates. Two patients were once reported as treatment success but were lost at the last yearly follow-up, thus excluded from the analysis. *MDR* Multidrug-resistant, SD Standard deviation, *DST* Drug susceptibility testing, *XDR* Extensively drug-resistant

^a^Adjusted by age, gender and study sites

## Discussion

The MDR-TB epidemic remains a global public health problem, compromising the achievements in TB control in the past three decades. Over a two-year enrolment by performing DST to all culture-positive TB cases in four geographically varied regions of China, covering a population of 12 million, 58 (5.0%) MDR-TB patients were detected from 1168 bacteriologically confirmed TB patients, which was far behind the estimation by the WHO. The results indicated that even with such a small amount of detected MDR-TB patients, most of them experienced major problems in obtaining timely MDR-TB diagnosis and appropriate treatment. More than half of patients had a delay longer than 90.0 days for receiving a DST report of MDR-TB, while only 32.8% were transferred to the up-level designated MDR-TB hospitals for MDR-TB treatment. Unfavourable outcomes occurred in 64.3% of diagnosed patients, resulting in a high risk of disease transmission in population as well as an increased risk of mortality.

The priorities of global MDR-TB control action ask for high-quality drug-susceptible TB treatment, rapid testing and detection of MDR/RR-TB, prompt access to effective treatment and proper care, minimized risk of disease transmission and enhanced financing. The proportion of participants with MDR-TB diagnosis delay above 90.0 days was 51.7% in this study, lower than the 81.0% reported in another Chinese study [8]. The difference might be explained by the delay caused by sending a positive mycobacterial culture to the provincial TB laboratory for DST in that study. Notably, 13.8% of the participants had a diagnosis delay over 180.0 days, almost the full duration of a drug-susceptible TB treatment course. To shorten the waiting time for MDR-TB diagnosis, rapid molecular diagnostics have been strongly recommended internationally [2]. As the country with the heaviest MDR-TB burden, India has made great efforts on the scale up of rapid molecular testing [18]. However, rapid molecular diagnostics weren’t frequently used in China, with an approximate proportion of 15% in 2018 [2], because of their high cost and out-of-pocket expenditures [19].

The poor accessibility of MDR-TB treatment even in detected MDR-TB patients also calls for immediate action. In South Africa, the treatment coverage for MDR-TB patients reached around 70% in 2018 [2], far above the accessibility shown in our study. The reason might be the high proportion of MDR-TB patients from our western study sites where accessibility to MDR-TB treatment was poorer than eastern regions [5]. Apart from geographic variations, the long waiting time for DST results is a probable cause leading to low coverage of MDR-TB treatment. In the non-MDR-TB treatment group, 15.4% of patients got their DST results after the completion of drug-susceptible TB treatment, posing a psychological barrier to motivate these patients to start a treatment course for MDR-TB. In addition, effective therapeutic effects of first-line drug regimen judged by clinicians or refusal of transferring to MDR-TB treatment made by patients were also the reasons causing low transfer rate to MDR-TB treatment. It indicated that health education of MDR-TB care should be enhanced for both clinicians and patients to improve their awareness.

A large proportion of patients ended up their treatment with loss to follow-up, treatment failure and incomplete treatment, thus amplifying the risk of MDR-TB transmission. It may help to explain the findings in previous studies that over 70% of detected MDR-TB patients in China were caused by primary transmission [20, 21]. Similar results were also observed in our study that 56.9% (33/58) of MDR-TB patients did not have previous TB treatment history. After being transferred to MDR-TB treatment, 57.9% of patients were lost to follow-up or did not adhere to MDR-TB regimen. Previous studies indicated that reasons for loss during referral were inconvenient transportation, poor adherence of patients or foreseeable heavy economic burden [10, 22]. Furthermore, poor coordination between different sections under China’s MDR-TB control program could be another reason, because the loss to follow-up occurred at the time of changing to outpatient treatment. To minimize the loss to follow-up and untreated cases, political commitment with financing should be prioritized and enhanced. The high cost for MDR-TB treatment [23, 24] made it both unaffordable and catastrophic for patients [10].

As expected, patients with MDR-TB but not receiving MDR-TB treatment regimen had a high proportion of unfavourable outcomes. Until the end of 2019, 27.6% of them were confirmed with TB relapse while 13.8% had died within two years after treatment completion. It’s an appalling but reasonable outcome due to the loss of the two most effective drugs [25], i.e. isoniazid and rifampicin, leaving only pyrazinamide and ethambutol as possible effective agents. Similar results were reported by other studies where MDR-TB patients with first-line TB treatment had high probability of TB relapse and death after the treatment completion [26, 27]. The possible explanation for the “treatment success” at the end of TB treatment might be the effect of pyrazinamide, an agent with strong sterilizing effect in the combination therapy [25, 28], and ethambutol. Another possible explanation is the lower proportion of mutations in *katG/inhA* loci in non-MDR-TB treatment group (62.9%). The inconsistent between phenotypic and genotypic drug resistance indicated isoniazid might be an effective drug for some patients, highlighting the necessity of repeating DST and molecular diagnostics. However, ineffective treatment would increase the risk of developing additional drug resistance [29]. Providing first-line TB treatment for MDR-TB patients will most certainly promote further resistance to pyrazinamide and ethambutol.

A strength of this study is featured by the up to 6-year follow-up for the cohort, which could add valuable information on the experiences of MDR-TB patients on diagnosis and treatment, especially those ended up with treatment failure, incomplete treatment and loss to follow-up. Our study also has some limitations. Firstly, though mycobacterial culture and DST were given to both smear-positive patients and smear-negative patients with typical pulmonary lesions on chest X-ray, the rest smear-negative TB patients without culture confirmation could lead to underestimation of total number of MDR-TB patients, even if it might be a very small amount. The reasons for not providing mycobacteria culture to all the smear-negative TB patients were the huge workloads and capacity constrains in designated county or district TB clinics, and the high uncertainty in diagnosing smear-negative TB patients. In this study, we only included clinically highly suspected smear-negative TB patients with typical pulmonary lesions on chest X-ray for sputum culture. Secondly, the small sample size limited the power of association analysis on unfavourable outcomes, though a two-year enrolment was given in four Chinese cities. Thirdly, treatment success in non-MDR-TB treatment group was still not well clarified. However, we believe that priority should be made for the provision of high-quality MDR-TB care for detected MDR-TB patients. Finally, patients remained in the treatment success at the last follow-up and those lost to follow-up were not verified by the bacteriological confirmation, which might lead to the underestimation of TB relapse. The high incidence of TB relapse in these patients should be enough to prove the ineffectiveness of treating MDR-TB patients with first-line TB drugs.

## Conclusions

It is crucial for China’s MDR-TB control program to establish high quality of care to all detected MDR-TB patients, especially in less developed regions. Our study showed that delayed MDR-TB diagnosis resulted in poor quality of MDR-TB care. Meanwhile, treating MDR-TB patients with a first-line drug regimen was found to be ineffective and led to a high incidence of unfavourable outcomes. It also constituted a risk for acquisition of additional drug resistance, especially in cases where a FQ was added to a failing regimen. The large proportion of loss to follow-up and untreated cases was a risk factor for increased disease transmission in population, as well as increased mortality among patients. Our findings highlight the urgency of strengthening patient management and financial support.

## Data Availability

The datasets used and analysed during the current study are available from the corresponding author on reasonable request.

## Abbreviations

MDR-TB: Multidrug-resistant tuberculosis
WHO: World Health Organization
RR: Rifampicin-resistant
DOTS: Directly observed treatment, short course strategy
DST: Drug susceptibility testing
*M.tb*: *Mycobacteria tuberculosis*
CDC: Center for Disease Control and Prevention
LJ: Loewenstein-Jenson
CTAB: Cetyl Trimethyl Ammonium Bromide
FQ: Fluoroquinolone
SLID: Second-line injectable drugs
pre-XDR: Pre-extensively drug-resistant
